# Numb has distinct function in lung adenocarcinoma and squamous cell carcinoma

**DOI:** 10.18632/oncotarget.25585

**Published:** 2018-06-29

**Authors:** Hajime Kikuchi, Jun Sakakibara-Konishi, Megumi Furuta, Eiki Kikuchi, Junko Kikuchi, Satoshi Oizumi, Yasuhiro Hida, Kichizo Kaga, Ichiro Kinoshita, Hirotoshi Dosaka-Akita, Masaharu Nishimura

**Affiliations:** ^1^ Department of Respiratory Medicine, Faculty of Medicine and Graduate School of Medicine, Hokkaido University, Sapporo, Japan; ^2^ Department of Respiratory Medicine, National Hospital Organization Hokkaido Cancer Center, Sapporo, Japan; ^3^ Department of Cardiovascular and Thoracic Surgery, Faculty of Medicine, Hokkaido University, Sapporo, Japan; ^4^ Department of Medical Oncology, Hokkaido University Graduate School of Medicine, Sapporo, Japan

**Keywords:** Numb, Notch1, non-small cell lung cancer, adenocarcinoma, squamous cell carcinoma

## Abstract

Some reports suggest that Numb is a potential tumor suppressor. However, its role in non-small cell lung cancer remains unclear. Non-small cell lung cancer comprises two major histological subtypes, adenocarcinoma and squamous cell carcinoma. To investigate the role of Numb in tumorigenesis of lung adenocarcinoma and squamous cell carcinoma, we firstly performed loss-of-function and gain-of-function assays. Moreover, Numb expression was investigated in surgically resected lung adenocarcinoma and squamous cell carcinoma tissues by immunohistochemistry and correlations with prognosis were analyzed. Numb suppressed the proliferation, migration, and invasion of adenocarcinoma cells and inhibited Notch signaling and epithelial-mesenchymal transition *in vitro*. Numb overexpression also inhibited subcutaneous adenocarcinoma tumor growth. In contrast, Numb promoted the proliferation, migration, and invasion of squamous cell carcinoma cells, but did not induce any consistent changes in Notch signaling. High Numb expression was associated with favorable prognosis in patients with lung adenocarcinoma, but not in those with squamous cell carcinoma. Collectively, our data demonstrate that Numb plays distinct roles in lung adenocarcinoma and squamous cell carcinoma. In lung adenocarcinoma, Numb impairs tumor growth and inhibits the Notch pathway and epithelial-mesenchymal transition, whereas in lung squamous cell carcinoma it may promote proliferation.

## INTRODUCTION

Lung cancer is a leading cause of cancer death worldwide, and despite remarkable advances in treatment, its incidence is still increasing [1, 2]. Non-small cell lung cancer (NSCLC) accounts for >80% of all lung cancers. It comprises two major histological types, adenocarcinoma (ADC) and squamous cell carcinoma (SCC). They are thought to originate from unique cells of origin, arise through different initiating oncogenic events, and involve the activation of distinct signaling pathways. KRAS or EGFR gene mutations induce a development of lung ADC in Alveolar type 2 or Clara cells, whereas genomic alteration of TP53, PTEN or SOX2 causes a development of lung SCC in basal, Clara or Alveolar type 2 cells [3–6]. Therefore, targeted therapeutic approaches and the assessment of acquired drug-resistance mechanisms based on histological subtypes of NSCLC would be required.

The Notch signaling pathway regulates many fundamental processes essential for normal development such as the control of cell differentiation, survival, proliferation, and angiogenesis. In mammals, there are four Notch receptors (Notch1, Notch2, Notch3, and Notch4) and two families of ligands, Jagged (JAG1, 2) and Delta-like ligands (DLL1, 3, 4) [7]. Numb was originally identified in Drosophila and is an evolutionarily conserved protein that plays a critical role in the control of asymmetric self-renewal, progenitor cell fate determination, cell adhesion, and migration [8]. Experimental evidence indicates that Numb inhibits Notch signaling [9, 10] and has the potential to function as a tumor suppressor. In esophageal squamous cell carcinoma and breast cancer, Numb overexpression suppresses tumor cell growth and epithelial-mesenchymal transition (EMT) by antagonizing Notch signaling, and loss of Numb expression is associated with poor prognosis [11, 12]. In contrast, it has been reported that Numb promotes cell proliferation and is correlated with poor prognosis in hepatocellular carcinoma [13]. These reports suggest that Numb might have different functions according to the type of malignancy or the histological subtype. Regarding NSCLC, one previous study demonstrated a loss of Numb expression and an inverse correlation between the levels of Numb and Hes1, a Notch target gene [14]. However, the role of Numb in tumorigenesis and the association between Numb expression and prognosis remains unclear.

In this study, we analyzed distinct function of Numb in lung ADC and SCC. We firstly modulated Numb expression to reveal the effect on proliferation, migration, invasion, and tumor formation. Secondly, we analyzed the expression of Numb in surgically resected lung ADC and SCC patient tissues by immunohistochemistry and evaluated the relationship of Numb expression with prognosis.

## RESULTS

### Effect of Numb inhibition on lung ADC and SCC cell proliferation, migration, and invasion

**Figure 1:**
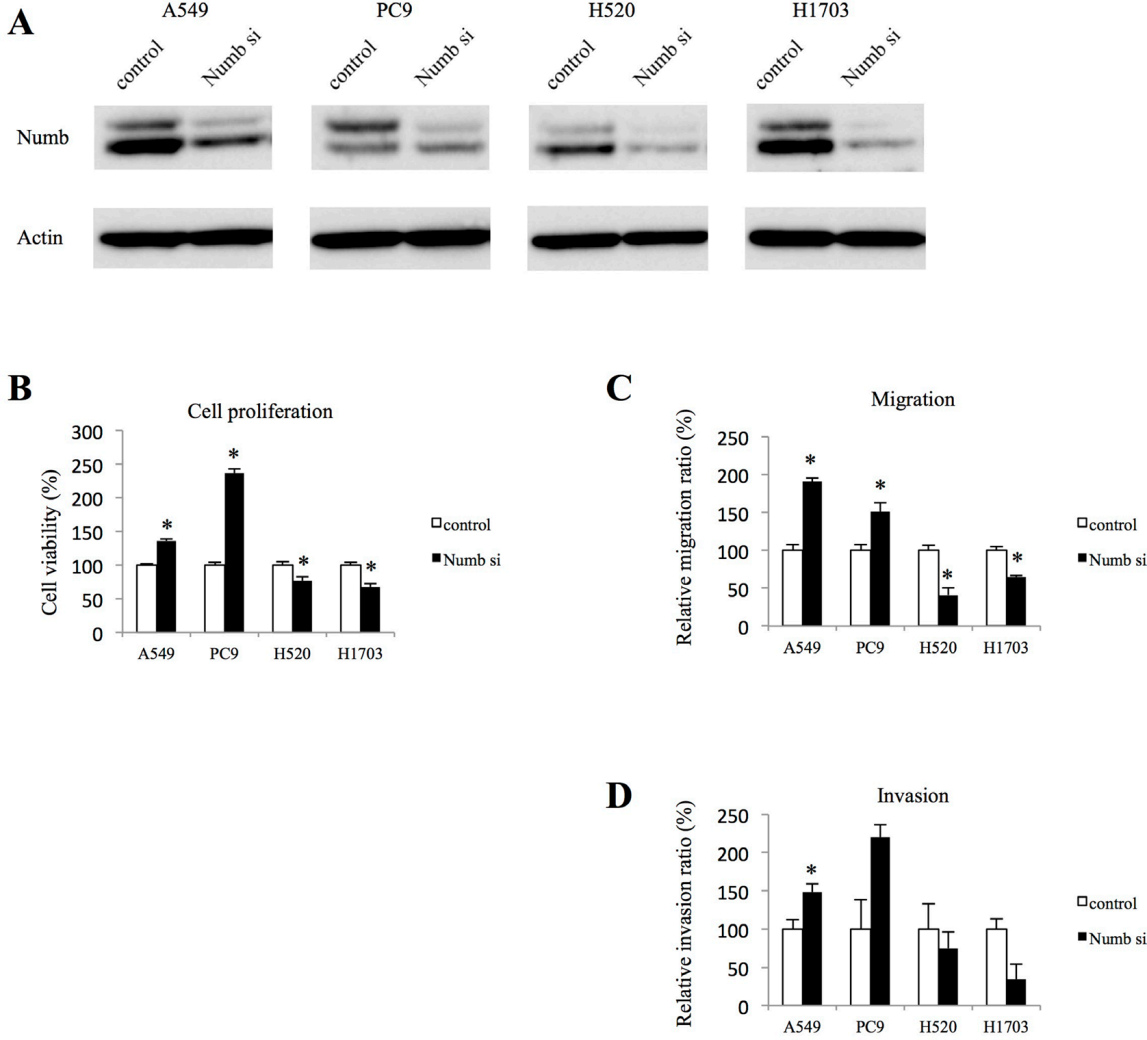
Effect of Numb knockdown on cell proliferation, migration, and invasion in lung adenocarcinoma and squamous cell carcinoma cell lines (**A**) Numb protein expression levels in A549, PC9, H520 and H1703 cells transfected with nonspecific or Numb-siRNA were measured by WB at 48 h after transfection. (**B**) Anchorage-independent cell growth was measured by MTT assays using 96-well plates with poly-HEMA coating at 72 h after nonspecific or Numb-siRNA transfection (*n* = 5, mean ± SEM). (**C**) Cells were plated in the upper chamber 48 h after transfection with nonspecific or Numb-siRNA. After incubation for 4 h, the number of migrated cells was counted in five random fields of view (*n* = 3, mean ± SEM). (**D**) Cells were plated in the upper chamber, which was pre-coated with Matrigel, 48 h after transfection with nonspecific or Numb-siRNA. After incubation for 24 h, the number of invaded cells was counted in five random fields of view (*n* = 3, mean ± SEM). ^*^Indicates *P* < 0.05. Numb si: Numb siRNA.

To investigate the role of Numb in lung ADC and SCC, lung ADC cell lines (A549, PC9) and SCC cell lines (H520, H1703) were transfected with Numb small interfering RNA (siRNA). After transfection, the Numb protein levels were downregulated in all cell lines (Figure 1A). We examined the effect of Numb knockdown on cell proliferation. Numb knockdown in ADC cells resulted in a significant increase in anchorage-independent proliferation when compared to that in control cells. Conversely, Numb knockdown inhibited anchorage-independent proliferation in SCC cells (Figure 1B). Moreover, Numb inhibition increased the number of migrating and invading ADC cells, whereas it suppressed SCC cell migration and invasion (Figure 1C and 1D).

### Effects of Numb on Notch signaling and EMT in lung ADC and SCC cells

**Figure 2:**
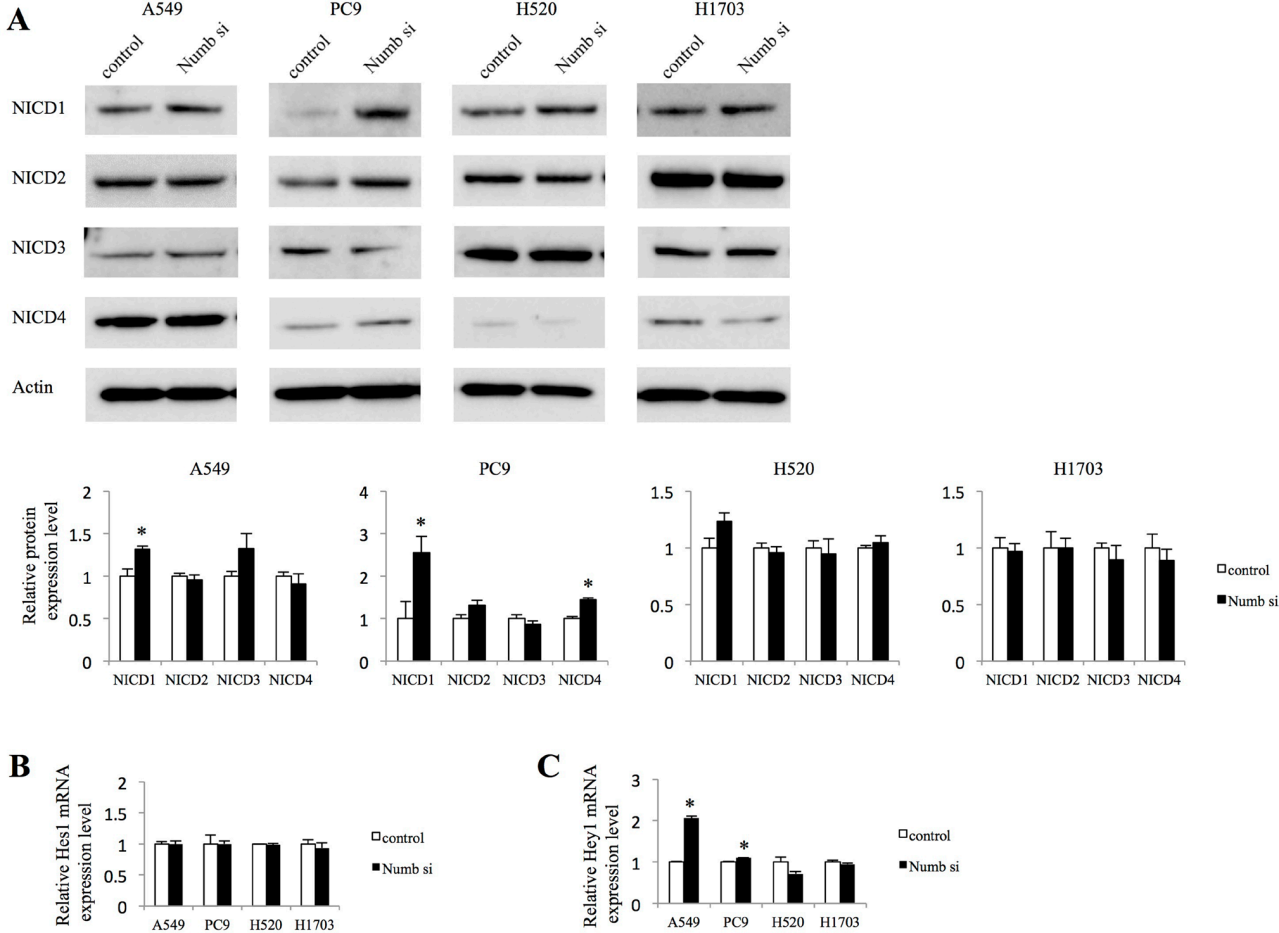
Effect of Numb knockdown on Notch signaling in lung adenocarcinoma and squamous cell carcinoma cell lines (**A**) NICD1, NICD2, NICD3, and NICD4 protein expression levels in A549, PC9, H520, and H1703 cells transfected with nonspecific or Numb-siRNA were measured by WB at 48 h after transfection (*n* = 3, mean ± SEM). (**B**, **C**) Fold-change expression of Notch target genes, Hes1 and Hey1, at 72 h after transfection with Numb-siRNA, relative to expression in the control (*n* = 3, mean ± SEM).

Because Numb has been reported to act as a repressor of the Notch pathway [9, 10], we investigated whether Numb inhibition affects Notch signaling. The expression of Notch intracellular domain (NICD) 1, NICD2, NICD3, and NICD4 and the Notch target genes, Hes1 and Hey1, were assessed by western blotting analysis (WB) and quantitative real-time polymerase chain reaction (qRT-PCR). Suppression of Numb with siRNA significantly increased the NICD1 protein levels. However, the difference in NICD1 expression between the control cells and the Numb siRNA transfected cells was marginal in lung ADC cells (Figure 2A). In contrast, Numb inhibition did not change NICD1 expression in lung SCC cells (Figure 2A). NICD2, NICD3, and NICD4 were not affected by inhibition of Numb, with the exception of increased NICD4 expression in PC9 cells transfected with Numb-siRNA (Figure 2A). The suppression of Numb did not affect Hes1 mRNA levels in any cell line (Figure 2B). In contrast, Numb knockdown augmented Hey1 mRNA levels in both ADC cell lines, but not in SCC cell lines (Figure 2C).

**Figure 3:**
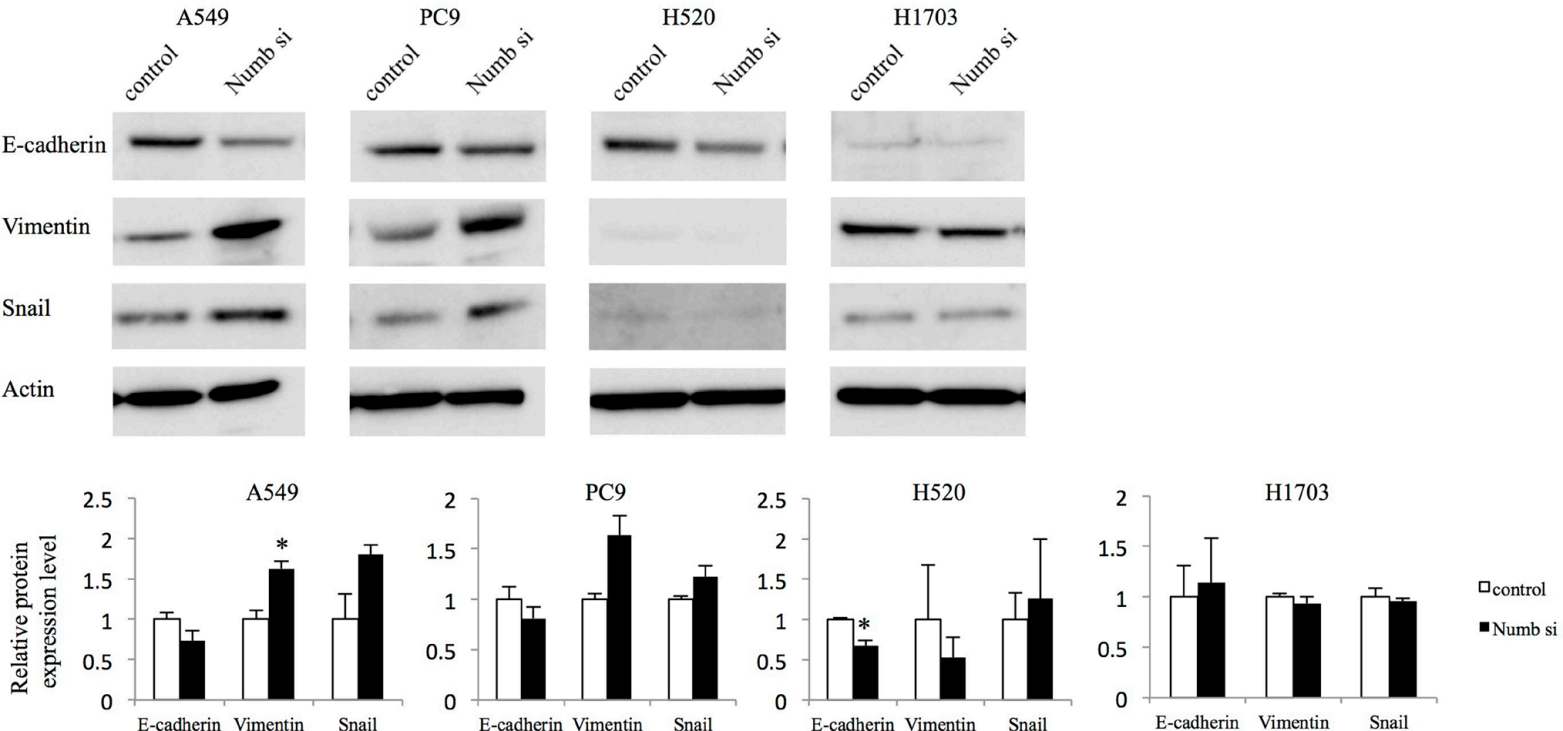
Effect of Numb knockdown on EMT in lung adenocarcinoma and squamous cell carcinoma cell lines EMT marker protein expression levels in A549, PC9, H520, and H1703 cells transfected with nonspecific or Numb-siRNA were measured by WB at 48 h post-transfection (*n* = 3, mean ± SEM). ^*^Indicates *P* < 0.05. Numb si: Numb siRNA.

Because it has been demonstrated that cancer cells undergo EMT, acquiring the ability to migrate and metastasize in several previous studies [15–18], we analyzed the expression of associated markers including E-cadherin, Vimentin, and Snail by WB (Figure 3). Numb knockdown marginally downregulated the protein levels of E-cadherin, compared to control expression levels in both ADC cell lines. Vimentin was upregulated significantly in A549 cells and tended to be upregulated in PC9. The expression of Snail tended to increase in both ADC cells. In H1703 cells, Numb knockdown did not affect the expression of E-cadherin, Vimentin, or Snail; however, downregulation of E-cadherin was observed and Vimentin was tended to be downregulated by Numb suppression inH520 cells.

### Effects of Numb overexpression on lung ADC and SCC cell proliferation, migration, and invasion

**Figure 4:**
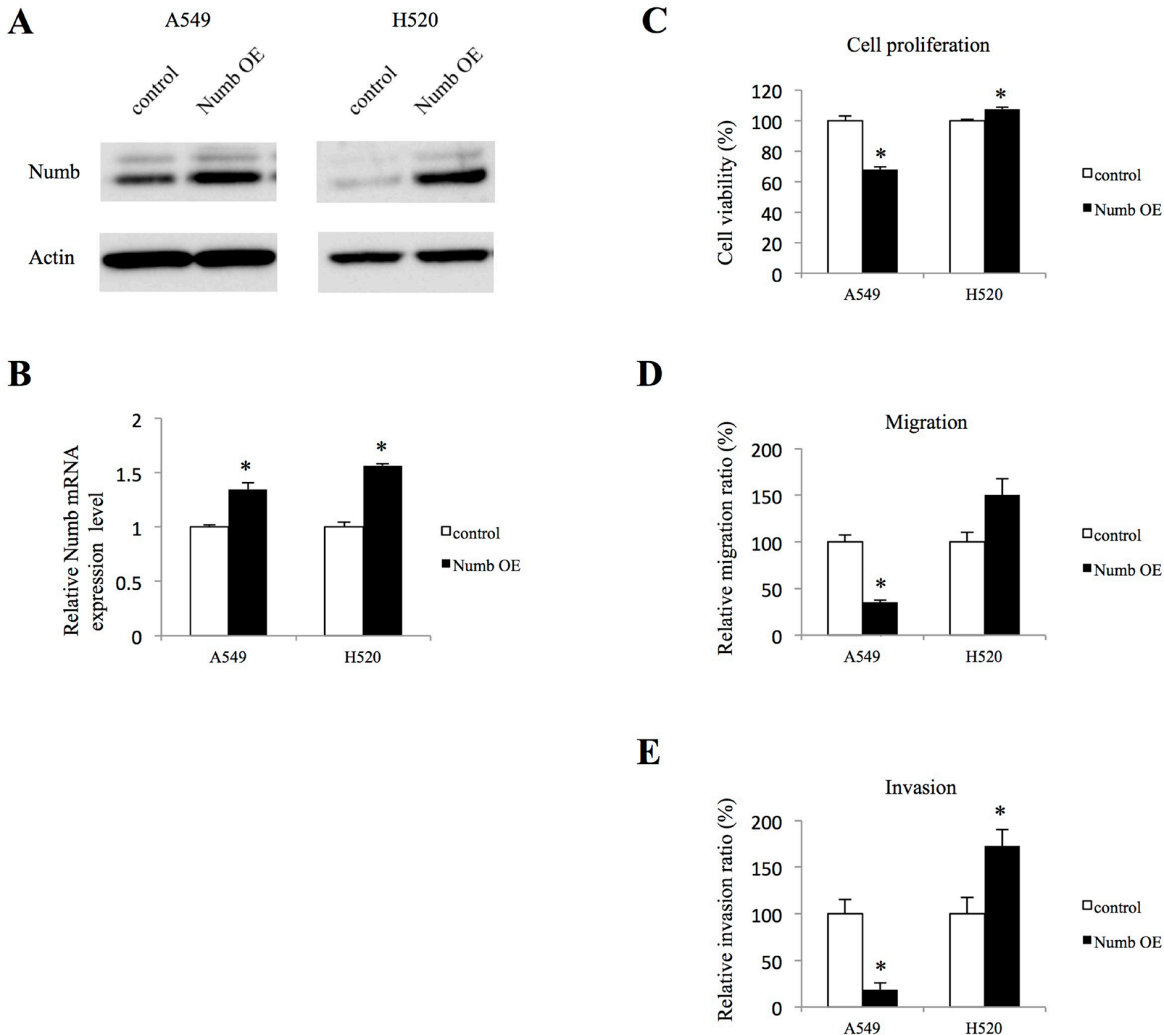
Effect of Numb overexpression on cell proliferation, migration, and invasion in A549 and H520 cells (**A**, **B**) Transfection of A549 and H520 cells with a Numb overexpression vector induced an increase in Numb expression, as detected by WB and qRT-PCR (*n* = 3, mean ± SEM). (**C**) Anchorage-independent cell growth was measured by MTT assays using 96-well plates with poly-HEMA coating at 72 h after seeding A549 and H520 cells transfected with empty control or Numb overexpression vector (*n* = 5, mean ± SEM). (**D**) A549 and H520 cells transfected with empty control or Numb overexpression vectors were plated in the upper chamber. After incubation for 4 h, the number of migrated cells was counted in five random fields of view (*n* = 3, mean ± SEM). (**E**) A549 and H520 cells transfected with empty control or Numb overexpression vectors were plated in the upper chambers pre-coated with Matrigel. After incubation for 24 h, the number of invaded cells was counted in five random fields of view (*n* = 3, mean ± SEM). ^*^Indicates *P* < 0.05. Numb OE: Numb overexpression.

To further confirm the role of Numb in lung ADC and SCC, A549 and H520 cells were transfected with a Numb expression vector, which resulted in significant upregulation at the protein and mRNA levels (Figure 4A and 4B). We also attempted the transfections in PC9 and H1703 cells but they failed. In A549 cells, Numb overexpression resulted in statistically significant inhibition of anchorage-independent proliferation when compared to those in control cells). Conversely, a modest increase in anchorage-independent proliferation was observed in Numb-overexpressing H520 cells (Figure 4C). Cell migration and invasion assays showed that Numb overexpression statistically significantly suppressed migration and invasion in A549 cells (Figure 4D and 4E). In contrast, Numb overexpression induced migration and invasion in H520 cells (Figure 4D and 4E).

### Effects of Numb overexpression on Notch signaling and EMT in lung ADC and SCC cells

**Figure 5:**
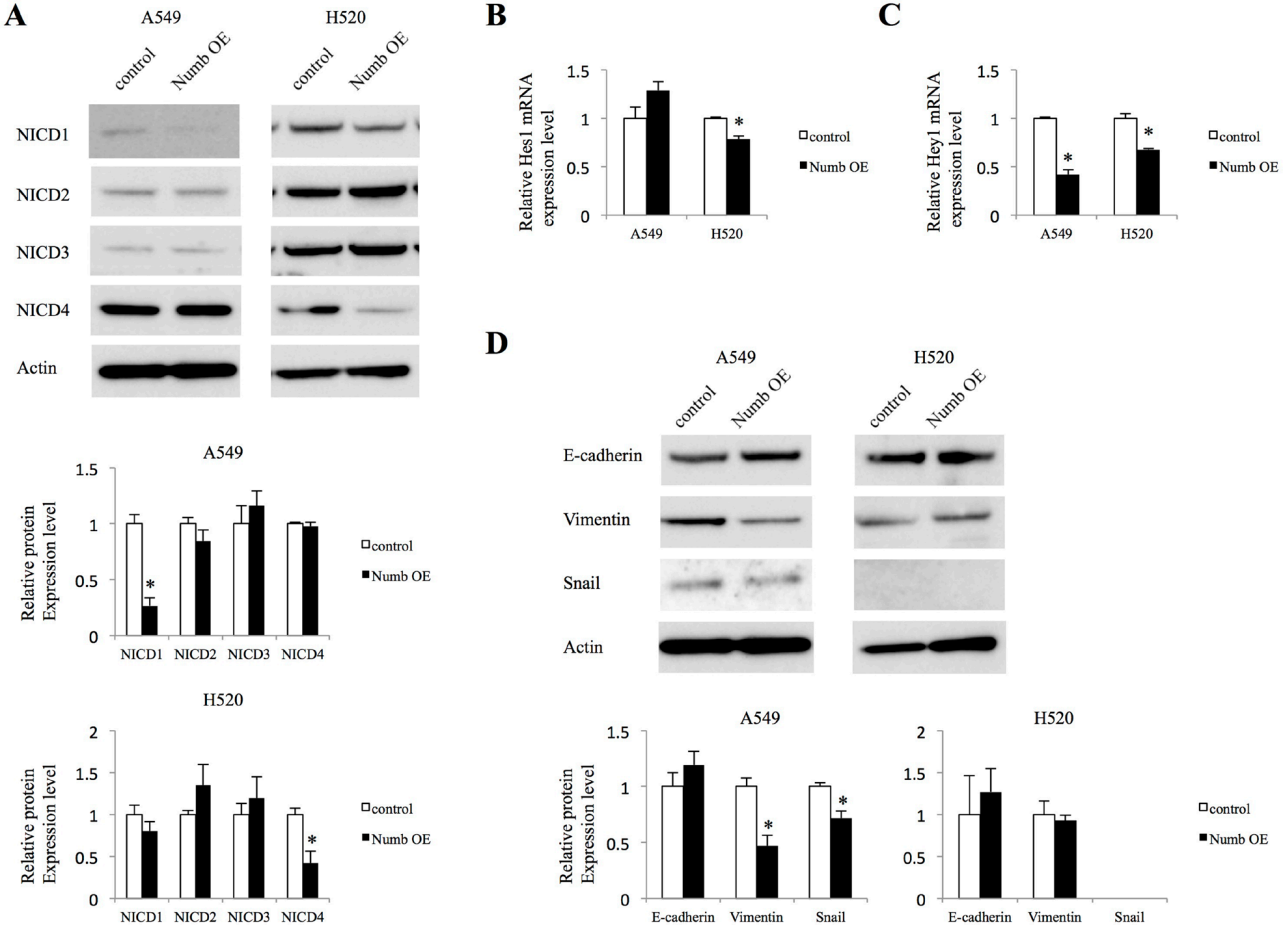
Effect of Numb overexpression on Notch signaling and EMT in A549 and H520 cells (**A**) Protein expression levels of NICD1, NICD2, NICD3, and NICD4 were analyzed in A549 and H520 cells transfected with empty control or Numb overexpression vector (*n* = 3, mean ± SEM). (**B**, **C**) Fold-change expression of Notch target genes Hes1 and Hey1 after transfection with Numb overexpression vector, relative to expression in the control (*n* = 3, mean ± SEM). (**D**) Protein expression levels of EMT markers were analyzed in A549 and H520 cells transfected with empty control or Numb overexpression vectors (*n* = 3, mean ± SEM). ^*^Indicates *P* < 0.05. Numb OE: Numb overexpression.

Next, we investigated whether Numb overexpression affects Notch signaling and EMT markers. In A549 cells, increased expression of Numb downregulated the NICD1 protein levels (Figure 5A). However, NICD2, NICD3, and NICD4 were not affected by Numb overexpression in these cells. We examined mRNA expression of Notch1, Notch2, and Notch3 to confirm the results of NICD protein expression in Numb overexpressing A549 cells. Notch1 mRNA was suppressed by Numb overexpression and Notch2 and Notch3 were not affected, which were consistent with the results of protein expression of NICD (Supplementary Figure 1A). In contrast, in H520 cells, increased Numb expression significantly downregulated the protein levels of NICD4, but not those of NICD1, NICD2, or NICD3 (Figure 5A). Hes1 mRNA levels were downregulated by Numb overexpression in H520 cells, but not in A549 cells (Figure 5B). Numb overexpression resulted in decreased mRNA levels of Hey1 in both A549 and H520 cells (Figure 5C). E-cadherin levels were moderately upregulated in A549 cells by overexpression of Numb, although not statistically significant. On the other hand, the protein levels of Vimentin and Snail were significantly downregulated in A549 cells when Numb was overexpressed (Figure 5D). Because expression difference of protein levels of EMT markers was too small in A549 cells, we evaluated mRNA expression. E-cadherin mRNA levels were moderately upregulated, although not statistically significant. Snail was downregulated significantly and Vimentin tended to be downregulated by Numb overexpression (Supplementary Figure 1B). These mRNA data in A549 cells by Numb overexpression were consistent with data about protein expression. We repeated the analysis of Snail expression when H520 cells were transfected with Numb control and overexpression vector. However, the expression of Snail was not observed (Figure 5D). Snail expression might be lost during the vector transfection.

### Effects of Numb overexpression on subcutaneous lung ADC cell tumor growth

**Figure 6:**
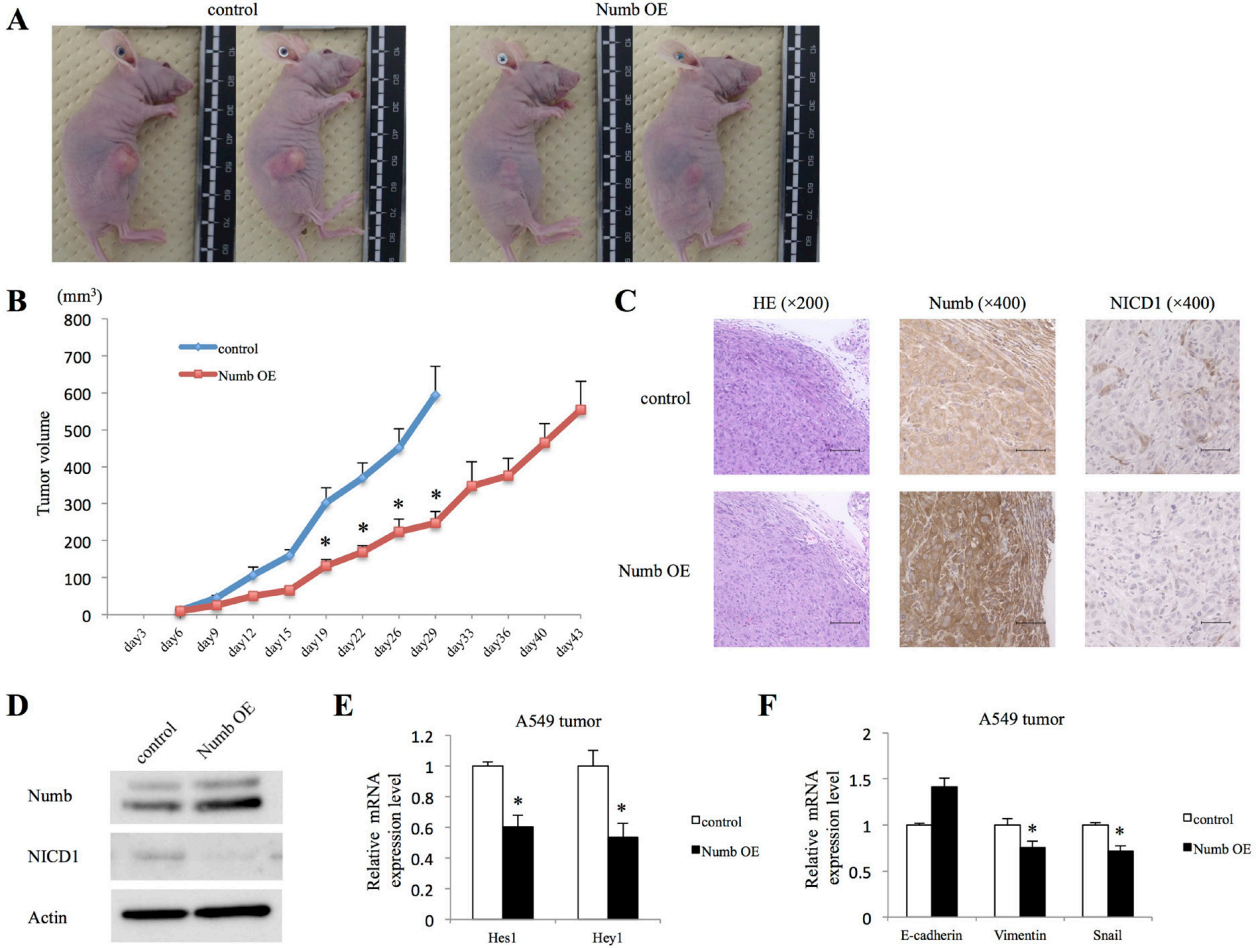
Effect of Numb overexpression on A549 subcutaneous tumor formation *in vivo* A549 cells transfected with empty control or Numb overexpression vectors were implanted into nude mice. (**A**) Photographs of representative tumors on day 30 after implantation are shown. (**B**) Summary of control and Numb overexpression tumor growth curves in nude mice showing the average tumor volume (*n* = 6, mean ± SEM). (**C**) IHC and (**D**) WB were performed to verify Numb overexpression and NICD1 downregulation in Numb-overexpressing tumors, compared to that in control tumors on day 15 after implantation. HE staining: scale bar = 100 μm, Numb and NICD1 IHC: scale bar = 50 μm. (**E**) Fold-change expression of Hes1 and Hey1 mRNA in Numb-overexpressing tumors, compared to that in control tumors on day 15 after implantation (*n* = 3, mean ± SEM). (**F**) Fold-change expression of E-cadherin, Vimentin, and Snail mRNA in Numb-overexpressing tumors, compared to that in control tumors on day 15 after implantation (*n* = 3, mean ± SEM). ^*^Indicates *P* < 0.05. Numb OE: Numb overexpression.

We then investigated whether Numb overexpression suppresses ADC tumor growth *in vivo*. Although we attempted to examine tumor proliferation of H520 lung SCC cells, those cells could not be implanted *in vivo*. Tumor volumes in nude mice implanted with Numb-overexpressing A549 cells were significantly smaller than those in of the paired corresponding control group (Figure 6A and 6B). Primary tumors were resected and histopathologic examination, after hematoxylin and eosin (HE) staining, was performed (Figure 6C). Subcutaneous tumors of the Numb-overexpression group expressed higher levels of Numb than those of the control group, based on Immunohistochemical analysis (IHC) and WB (Figure 6C and 6D). Furthermore, as expected, NICD1 expression in tumors from the Numb-overexpression group was downregulated compared to that in the control group. Next, we investigated the effects of Numb overexpression on Notch downstream genes and EMT markers in A549 subcutaneous tumors. Numb overexpression decreased both Hes1 and Hey1 mRNA levels (Figure 6E). E-cadherin levels were moderately upregulated in A549 subcutaneous tumors by overexpression of Numb, but not statistically significant. Vimentin and Snail mRNA levels were significantly downregulated in Numb overexpressing A549 subcutaneous tumors (Figure 6F). These data in A549 subcutaneous tumors were consistent with those recorded for the *in vitro* Numb overexpressing experiment.

### Numb expression and lung ADC and SCC prognosis

**Figure 7:**
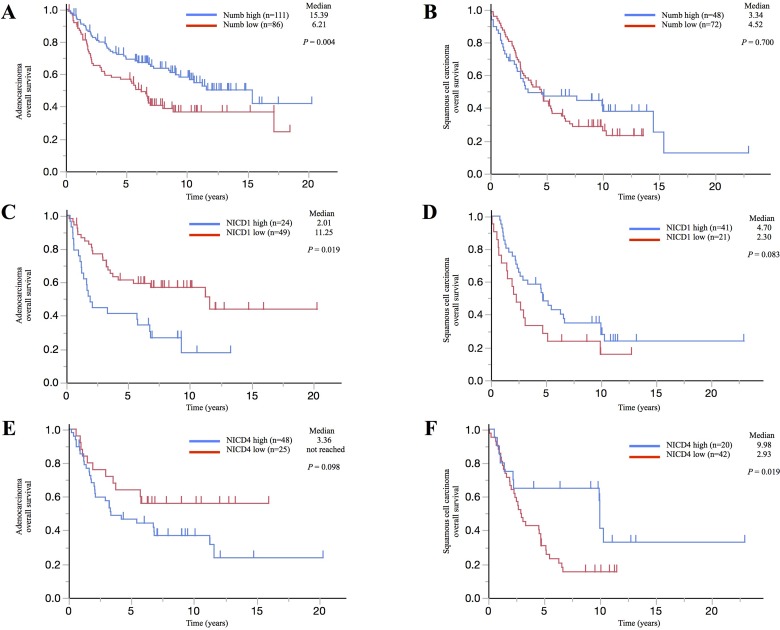
Correlation between Numb, NICD1, or NICD4 expression and prognosis in patients with lung adenocarcinoma or squamous cell carcinoma Kaplan–Meier survival curves illustrating overall survival in (**A**, **C**, **E**) lung ADC and (**B**, **D**, **F**) lung SCC patients with surgically resected tumors. Survival curves were stratified by the expression of (A, B) Numb, (C, D) NICD1, or (E, F) NICD4.

We evaluated the relationship of Numb expression with patients’ prognosis in surgically resected ADC or SCC tumor tissues by IHC. Clinicopathological characteristics of cohort1 and cohort2 are shown in Supplementary Table 1. To provide more accurate estimates of the overall effect of Numb expression in patients with lung ADC or SCC, we combined individual patient data in the cohort 1 and cohort 2 for an exploratory analysis of overall survival. Numb staining patterns in NSCLC tumors and normal bronchial and alveolar cells are shown in Supplementary Figure 2A and 2B. Specimens with enhanced staining relative to the median positive rate were classified into the high Numb expression group for each cohort. In ADC patients, high Numb expression significantly correlated with better overall survival (*P* = 0.004) (Figure 7A). However, in SCC patients, Numb expression was not significantly associated with overall survival (*P* = 0.700) (Figure 7B).

Moreover, we estimated NICD1 and NICD4 expression in cohort 1. NICD1 and NICD4 staining patterns in NSCLC tumors and normal bronchial and alveolar cells are shown in Supplementary Figure 3A and 3B. Cases were classified as performed for Numb expression. ADC patients with high NICD1 expression experienced significantly shorter survival compared to those with low NICD1 expression (*P* = 0.019), whereas SCC patients with high NICD1 expression tended to experience longer survival compared to those with low NICD1 expression (*P* = 0.083) (Figure 7C and 7D). Additionally, ADC patients with high NICD4 expression tended to experience shorter survival compared to those with low NICD4 expression (*P* = 0.098), whereas SCC patients with high NICD4 expression experienced significantly longer survival compared to those with low NICD4 expression (*P* = 0.019) (Figure 7E and 7F). Analysis of expression patterns showed that there is a significant inverse correlation between Numb and NICD1 expression levels in lung ADC, but not in SCC (Table 1). In contrast, there was no statistically significant association between Numb and NICD4 expression levels in either lung ADC or SCC (data not shown).

**Table 1:** Relationship between the expression of Numb and NICD1 in lung adenocarcinoma and squamous cell carcinoma

	Lung ADC^a^			Lung SCC^b^		
	NICD1^c^ expression			NICD1 expression		
	Low	High	*P*	Low	High	*P*
**Numb expression**						
Low	14	15	0.010	12	26	0.784
High	35	9		9	15	

^a^adenocarcinoma.

^b^squamous cell carcinoma.

^c^Notch1 intracellular domain.

## DISCUSSION

In this study, Numb was shown to have a tumor suppressive role, inhibiting the Notch pathway and EMT in lung ADC. We also discovered that Numb is a promising prognostic marker for patients with lung ADC. Meanwhile, *in vitro* studies demonstrated that Numb might be an activator of tumorigenesis in lung SCC cells, although Numb expression was not associated with overall survival in lung SCC patients. To our knowledge, this is the first study to clarify the distinct oncogenic role of Numb in lung ADC and SCC.

It was demonstrated that Numb can have both tumor promoting and inhibiting functions and those opposite effects of Numb might depend on tumor types [11–13]. Moreover, Numb was previously reported to negatively regulate Notch1 by promoting Notch1 ubiquitination and degradation of NICD1 [9, 10] and biological effects of Notch activity also seems to be tissue specific. We previously showed that the inhibition of Notch signaling by a γ-secretase inhibitor, which is a Notch inhibitor, suppresses the growth of NSCLC [19]. In lung ADC, Notch1 was reported to be an independent poor prognostic factor, but not in lung SCC [20]. This is in agreement with our results. In contrast, loss-of-function mutations in Notch receptors were found in cutaneous and lung SCC [21]. Based on previous reports, we hypothesized that Numb might have opposite functions in lung ADC and SCC through antagonizing the Notch pathway. First of all, we found that Numb inhibits tumor proliferation, migration, and invasion of lung ADC cells in this study. Furthermore, Numb downregulated the Notch pathway *in vitro* and *in vivo* in lung ADC cells and there was an inverse correlation between Numb and NICD1 expression levels in surgically resected lung ADC tumors. On the other hand, Numb promoted cell proliferation, migration, and invasion of lung SCC cells in this study. Although consistent changes in Notch signaling were not found in Numb-knockdown SCC cells, NICD4 and downstream genes, Hes1 and Hey1, were downregulated in Numb-overexpressing H520 cells. Moreover, NICD4 was a relative poor prognostic factor in ADC and was a comparatively better prognostic factor in lung SCC. Because most previous studies have assessed the functional consequences of interactions between NICD1 and Numb [9, 10, 14, 22, 23], the interaction between other Notch receptors and Numb is unclear. Although Notch4 acts as tumor activator in several tumors [24], it has been reported that Notch4 mRNA or protein expression is not significantly correlated to overall survival in both lung ADC and SCC [20, 25] and the role of Notch4 in lung SCC remains unclear. However, our data suggested that Notch4 might function as a tumor suppressor in lung SCC and as a tumor activator in lung ADC similar with Notch1. Moreover, our results indicate that Numb might induce switch to oncogenic or tumor suppressor signaling through different Notch receptor which is dependent on the tumor subtype.

The Notch pathway was found to be a key regulator of EMT [26–29]. It has been reported that Notch1 induces Snail expression in immortalized endothelial cells *in vitro* [26] and that overexpression of NICD1 inhibits E-cadherin expression and induces EMT in ovarian cancer cells [28]. Although mesenchymal markers, Snail and Vimentin, were affected by Numb, the expression of E-cadherin only marginally changed in lung ADC cells in this study. E-cadherin is thought to be a dominant mediator of cell interactions, the loss of which results in a switch to mesenchymal phenotype [15, 16]. However, it was reported that E-cadherin inhibition is not necessarily required for cell motility and invasion in breast cancer cells [30]. Even though incomplete regulation of E-cadherin was observed in our study, our results suggested that Numb controls migration and invasion by modulating EMT and Notch pathway. This corroborates the findings of other reports on esophageal squamous cell carcinoma and breast cancer [11, 12]. Meanwhile, Numb promoted migration and invasion in lung SCC cells in this study. Nevertheless, the protein levels of E-cadherin, Vimentin, and Snail did not significantly change. It was reported that N-cadherin or Claudin-1 promotes the migration of ovarian cancer cells without the induction of complete EMT [31], suggesting that other molecules might contribute to Numb-induced migration and invasion in lung SCC; such pathways were not assessed in the current study.

In addition to inhibiting the Notch pathway, Numb exhibits diverse functions such as interacting with Itch to ubiquitinate Gli1, with Mdm2 to hamper the ubiquitination of p53 and with cadherin and integrins to control cell adhesion and migration [32–34]. Although the present study observed interactions between Numb and the Notch pathway in lung ADC, the contribution of the other pathways to the tumor suppressive role of Numb requires further investigation. Whereas, it has been reported that Numb promotes cell proliferation and cell cycle of hepatocellular carcinoma, inhibiting p21 and modulating CDK4 and SKP2 [13, 35]. Numb might interact with cell cycle molecules to promote the growth of lung SCC, although we have not yet investigated this.

In conclusion, this is the first study analyzing the distinct roles of Numb in lung ADC and SCC, although there are several limitations of this study, including a limited number of cell lines and tissue samples. This study highlights the clinical importance of thoroughly understanding how Numb diversely contributes to different types of cancer, especially with regard to tumor histopathology, and provides compelling evidence that Numb is a potential novel therapeutic target for lung ADC.

## MATERIALS AND METHODS

### Cell lines

We used two lung adenocarcinoma cell lines (A549, PC9) and two lung squamous cell carcinoma cell lines (H520, H1703). A549, H520, and H1703 were obtained from the American Type Culture Collection. PC9 was obtained from the European Collection of Authenticated Cell Cultures. Cell lines were maintained in RPMI supplemented with 10% fetal bovine serum (FBS) at 37° C in a humid environment of 5% CO_2_.

### Antibodies and western blotting analysis

Primary antibodies targeting Numb (1:1000 dilution; ab14140, Abcam, Cambridge, UK), NICD1 (1:1000 dilution; #3608, Cell Signaling, Danvers, MA, USA), NICD2 (1:5000 dilution; #5732, Cell Signaling, Danvers, MA, USA), NICD3 (1:500 dilution; ABP-PAB-10683, Allele Biotechnology, San Diego, CA, USA), NICD4 (1:1500 dilution; #2423, Cell Signaling, Danvers, MA, USA), E-cadherin (1:200 dilution; sc-7870, Santa Cruz Biotechnology, Santa Cruz, CA, USA), Vimentin (1:200 dilution; V6630, Sigma-Aldrich, St. Louis, MO, USA), Snail (1:1000 dilution; #3879, Cell signaling, Danvers, MA, USA) were used. All analyses included staining with Ponceau S, which confirmed that protein loading was equal. Band intensity was demonstrated by quantitative densitometric analysis using National Institutes of Health (NIH) ImageJ Ver1.49 software (NIH, Bethesda, MD, USA). Standardization was performed by measuring actin in the same blots with an anti-actin antibody (1:1500 dilution; A2066, Sigma-Aldrich, St. Louis, MO, USA).

### Quantitative real-time RT-PCR

Total RNA was isolated using the RNeasy Mini Kit (Qiagen, Valencia, CA, USA) following the manufacturer's instructions. RNA was reverse transcribed into complementary DNA (cDNA) using TaqMan reverse transcription reagents with random hexamers obtained from Applied Biosystems (Waltham, MA, USA). Expression of Numb, Notch1, Notch2, Notch3, Hes1, Hey1, E-cadherin, Vimentin, Snail and GAPDH mRNA was determined by performing qRT-PCR with the ABI Prism 7900HT Sequence Detection System (Applied Biosystems, Waltham, MA, USA) according to the manufacturer's instructions. Numb, Notch1, Notch2, Notch3, Hes1, Hey1, and GAPDH reagents were obtained from Applied Biosystems and E-cadherin, Vimentin, and Snail reagents were obtained from Hokkaido System Science Co., Ltd (Sapporo, Hokkaido, JPN). The mean relative expression levels were compared to the expression of an internal control (GAPDH). All PCR amplifications were performed using a MicroAmp optical 96-well reaction plate with a TaqMan Fast Universal PCR Master Mix and with the TaqMan Gene Expression Assay kit (Applied Biosystems, Waltham, MA, USA).

### Small interfering RNA transfection

A549, PC9, H520, and H1703 cells were seeded at a density of 1.5 × 10^5^/well into 6-well plates the day before transfection. The Numb-siRNA sequence is ON-TARGET plus SMART pool L-015902-00 obtained from GE HealthCare Dharmacon (Lafayette, CO, USA). Cells were transfected with 50 pmol siRNA in Opti-MEM medium (Invitrogen, Waltham, MA, USA) using 50 μl Lipofectamine RNAiMAX (Invitrogen, Waltham, MA, USA). To confirm the efficiency of siRNA transfection, Numb protein expression levels were measured by WB at 48 h after transfection. Nonspecific siRNAs against the target sequence, ON-TARGET plus Non-targeting pool D-001810-10-05 (GE HealthCare Dharmacon, Lafayette, CO, USA), were used as controls.

### Numb overexpression

The human cDNA-ORF clone of the Numb gene (Numb-ORF plasmid), empty control vector (pCMV6-entry), and transfection reagent Turbofectin8.0 were purchased from OriGene Technologies, Inc. (Rockville, MD, USA). A549 and H520 cells were divided equally into two groups as follows: Numb overexpression (transfected with Numb-ORF plasmid) and control (transfected with pCMV6-entry). The day before transfection, cells were seeded at a density of 2 × 10^5^/well into 6-well plates. Cells were transfected with 2 μg Numb-ORF plasmid or vector in serum-free medium Opti-MEM I (Thermo Fisher Scientific, Waltham, MA, USA) using 12 μl Turbofectin8.0. After 24 h, the transfected cells were diluted at 1:10 into 10-cm dishes and the culture medium was replaced with complete medium containing G418 (1000 μg/ml). Stable clones were obtained after screening using G418.

### Cell proliferation assays

Anchorage-independent cell growth was measured by MTT assays using 96-well plates with poly-HEMA coating at 72 h after Numb-siRNA transfection or at 72 h after seeding Numb-overexpressing cells. MTT assays were performed according to the manufacturer's recommendation (Thermo Fisher Scientific, Waltham, MA, USA), and the absorption of light was determined at 560 nm using a microplate reader (Varioskan Flash; Thermo Fisher Scientific, Waltham, MA, USA).

### Migration and Invasion assay

Cell migration and invasion assays were performed using 24-well Transwell plates pre-coated without or with Matrigel (BD Biosciences, Franklin Lakes, NJ, USA). Then, cells were plated in the upper chamber with culture medium containing 0.1% FBS. The lower chamber had culture medium containing 10% FBS. For migration assays or invasion assays, cells were incubated for 4 or 24 h, respectively, and the membrane was stained using the Diff-Quik technique. The number of migrated or invaded cells was counted in five random fields of view using a CKX 41 microscope (Olympus, Tokyo, Japan).

### *In vivo* tumorigenicity

All animal husbandry and experiments were performed under protocols approved by the Institutional Animal Care Committee at Hokkaido University School of Medicine. A549 cells transfected with empty control or Numb overexpression vectors (1.0 × 10^6^ cells) were diluted in 200 μl phosphate-buffered saline and injected subcutaneously into the right posterior leg of athymic, 5-week-old, female nude mice (nu+/nu+). Each group consisted of six mice. The tumors were then measured twice/week using digital calipers. Tumor volume (TV) was determined using the formula TV = (length) × (width) × (height)/2 [36]. Some tumors were resected on day 15 for WB and IHC.

### Tumor specimens

Cohort 1 included 135 consecutive patients primary tumor specimens, with adequate archival data, obtained during radical surgery at Hokkaido University Medical Hospital between 1982 and 1994. The pathological stage (pStage) was based on the 4th edition of the American Joint Committee on Cancer guidelines for postoperative tumor-lymph node-metastasis (TNM) classification. Cohort 2 included 182 consecutive patients; from these individuals, a tissue microarray of primary tumor specimens was obtained during radical surgery at Hokkaido University Medical Hospital between 1996 and 2004. pStage was reclassified according to the 7th version of the TNM staging system. Informed consent was obtained from all patients.

### Immunohistochemical analysis

IHC was performed as previous described [37]. Numb NICD1 and NICD4 were detected by IHC with a rabbit polyclonal anti-Numb antibody (1:500 dilution; ab14140, Abcam, Cambridge, UK) a rabbit monoclonal anti-NICD1 antibody (1:400 dilution; #3608, Cell Signaling, Danvers, MA, USA) and a rabbit polyclonal anti-NICD4 antibody (1:200 dilation; ab199295, Abcam, Cambridge, UK). Normal bronchial epithelial cells were used as positive controls for Numb NICD1 and NICD4 expression. Once processed by γ-secretase, NICD1 and NICD4 are released from the plasma membrane and translocates to the nucleus where they activates target gene transcription. Therefore, membrane staining only was considered negative and cytoplasm and/or nuclear staining were considered positive for the evaluation of activated Notch1 and Notch4 expression [14, 20]. Tumor cells were assessed from five random and non-overlapping fields (100 tumor cells per field, total of 500 tumor cells per specimen). ‘Moderate’ staining intensity was defined as that which was similar to the positive control. ‘Weak’ staining was less intense than that of the positive control. ‘Strong’ staining had a higher intensity than that of the positive control. Since there are few previous reports on Numb IHC, the definition of positive expression for Numb IHC is not standardized. According to previous studies, NICD1 positivity was defined as a moderate-to-strong staining intensity [14, 20]. In the present study, Numb positivity was defined as a moderate-to-strong staining intensity as NICD1. NICD4 was also defined as a moderate-to-strong staining intensity. IHC evaluation was performed independently by two investigators (H.K. and M.F.). In the case of a discrepancy, the final results were decided by consensus.

### Statistical analysis

Significant differences in the means between two samples were analyzed using Student's *t* tests. Correlation between the expression of Numb and Notch1 was analyzed using a Fisher's exact test. Survival curves were estimated using the Kaplan–Meier method, and differences in survival distributions were evaluated by performing a log-rank test. The level of significance was set at *P* < 0.05. Statistical analyses were performed using JMP software (JMP^®^ Pro 11.0.0; SAS Institute Inc, USA).

## SUPPLEMENTARY MATERIALS FIGURES AND TABLE



## Abbreviations

NSCLC: Non-small cell lung cancer
ADC: adenocarcinoma
SCC: squamous cell carcinoma
EMT: epithelial-mesenchymal transition
siRNA: small interfering RNA
NICD: Notch intracellular domain
WB: western blotting analysis
qRT-PCR: quantitative real-time polymerase chain reaction
HE: hematoxylin and eosin
IHC: Immunohistochemical analysis
FBS: fetal bovine serum
NIH: National Institutes of Health
cDNA: complementary DNA
TV: Tumor volume
