# Supplementary material for: Numb has distinct function in lung adenocarcinoma and squamous cell carcinoma
Source: Oncotarget. 2018 Jun 29;9(50):29379–91. doi: 10.18632/oncotarget.25585 (PMC6047666; doi:10.18632/oncotarget.25585)
Supplement: Supplementary file 1 [file oncotarget-09-29379-s001.pdf]

Numb has distinct function in lung adenocarcinoma and squamous cell carcinoma

SUPPLEMENTARY MATERIALS

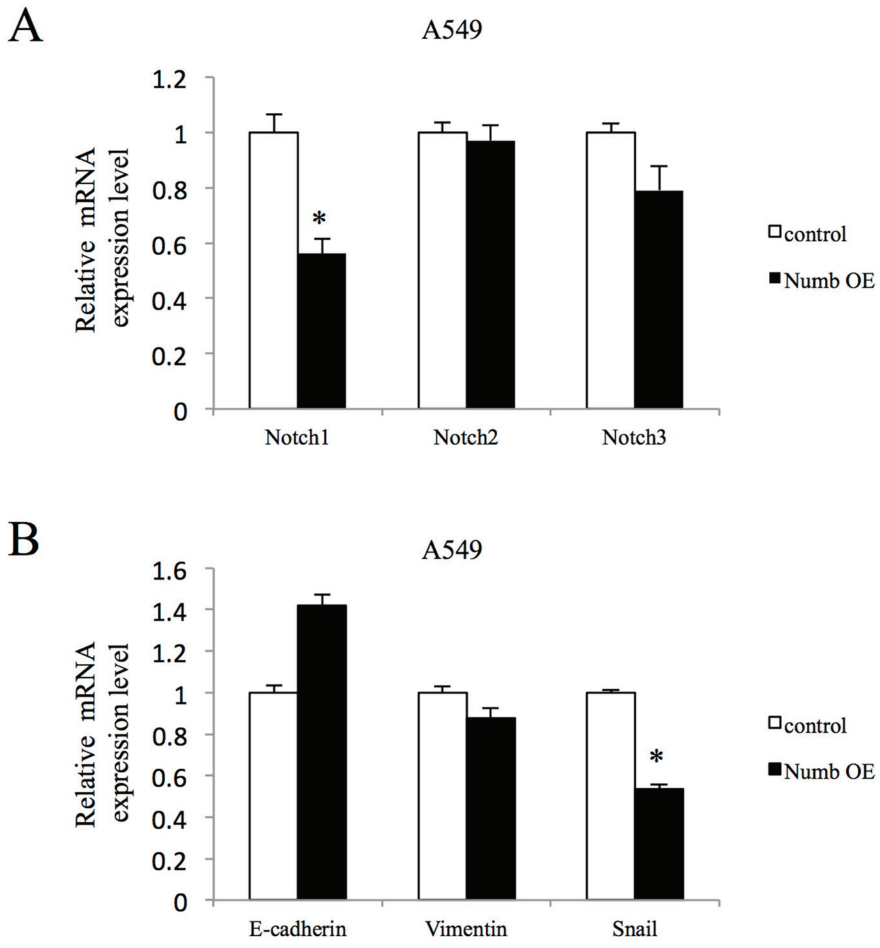

Supplementary Figure 1: Notch1–3 and EMT maker mRNA expression in Numb overexpressing A549 cells. (**A**) Fold-change in the levels of Notch1, Notch2, and Notch3 mRNA after transfection with Numb overexpression vector relative to the control ($n = 3$, mean ± SEM). (**B**) Fold-change in the levels of E-cadherin, Vimentin, and Snail mRNA after transfection with Numb overexpression vector relative to the control ($n = 3$, mean ± SEM). *Indicates $P < 0.05$. Numb OE: Numb overexpression.

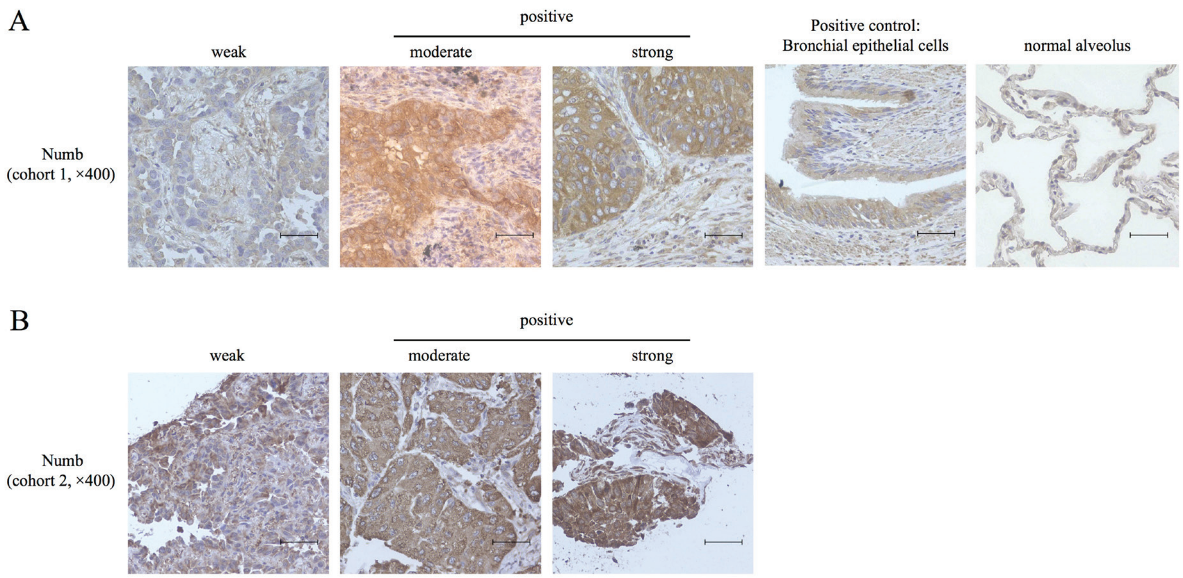

Supplementary Figure 2: Numb expression in non-small cell lung cancer tissues. (**A**, **B**) Immunohistochemical Numb staining patterns for the two cohorts are shown. NSCLC specimens were stained with anti-Numb antibody. Scale bar = 50 μm.

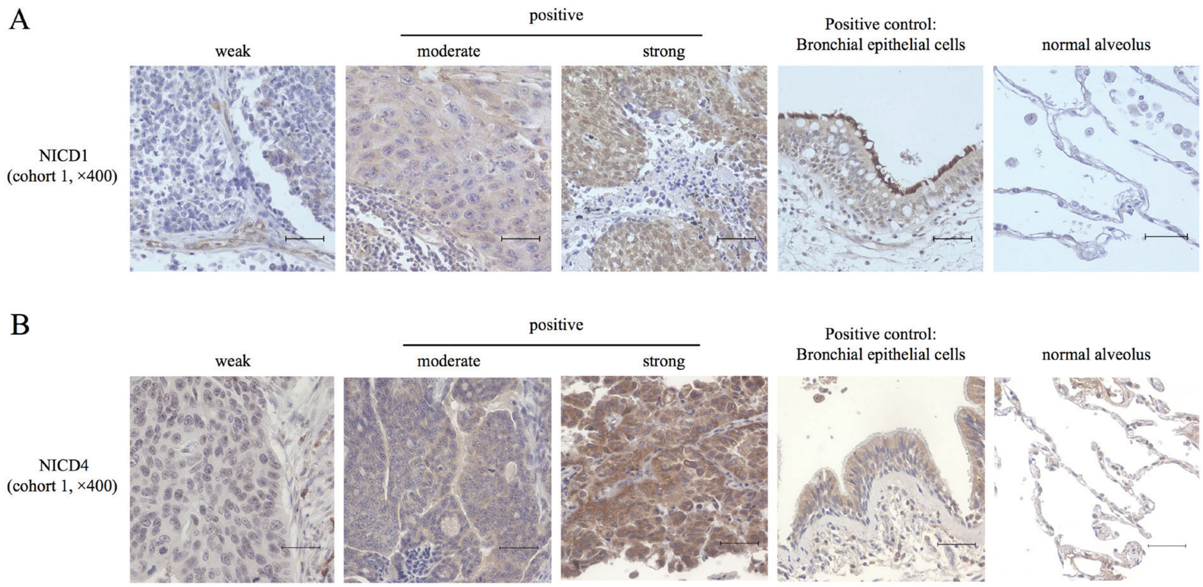

Supplementary Figure 3: NICD1 and NICD4 expression in non-small cell lung cancer tissues. (**A**) Immunohistochemical NICD1 staining patterns are shown for cohort 1. NSCLC specimens were stained with anti-NICD1 antibody. (**B**) Immunohistochemical NICD4 staining patterns are shown for cohort 1. NSCLC specimens were stained with anti-NICD4 antibody. Scale bar = 50 μm.

Supplementary Table 1: Clinicopathological characteristics of cohort1 and cohort2

	cohort1	cohort2
**Characteristics**	**No. of Patients**	**No. of Patients**
**Age (years)**	65 (31–85)	67 (30–92)
**Sex**		
Male	91	110
Female	44	72
**Histology**		
ADC[a]	73	124
SCC[b]	62	58
**pStagec**		
I	82	106
II	9	33
III	42	41
IV	2	2

[a]Adenocarcinoma.
[b]Squamous cell carcinoma.
[c]Pathological disease stage.
